# Cardiovascular, anthropometric, metabolic and hormonal profiling of normotensive women with polycystic ovary syndrome with and without biochemical hyperandrogenism

**DOI:** 10.1007/s12020-021-02648-7

**Published:** 2021-02-22

**Authors:** Małgorzata Kałużna, Tomasz Krauze, Katarzyna Ziemnicka, Katarzyna Wachowiak-Ochmańska, Jolanta Kaczmarek, Adam Janicki, Andrzej Wykrętowicz, Marek Ruchała, Przemysław Guzik

**Affiliations:** 1 Department of Endocrinology, Metabolism and Internal Diseases, Poznan University of Medical Sciences, Poznan, Poland; 2 Endocrinology, Metabolism and Internal Diseases Ward, Heliodor Swiecicki University Hospital, Poznan, Poland; 3 Department of Cardiology - Intensive Therapy, Poznan University of Medical Sciences, Poznan, Poland; 4 Central Laboratory, Heliodor Swiecicki University Hospital, Poznan, Poland

**Keywords:** Blood pressure, Cardiovascular function, Polycystic ovary syndrome (PCOS), Sex hormones, Hyperandrogenism, N-terminal of the prohormone of B-type natriuretic peptide (NT-proBNP)

## Abstract

**Purpose:**

Women with polycystic ovary syndrome (PCOS) present with or without biochemical hyperandrogenism (HAPCOS or non-HAPCOS, respectively). Cardiometabolic and hormonal abnormalities have been reported in women with PCOS, particularly those with hypertension. However, no direct comparison between normotensive (blood pressure <140/90 mmHg) patients with HAPCOS and non-HAPCOS has been made. This study compared different cardiovascular (CV), anthropometric, metabolic and hormonal features between normotensive patients with HAPCOS and non-HAPCOS and healthy women.

**Methods:**

We consecutively recruited 249 normotensive patients with PCOS and 85 healthy eumenorrheic women to a case-control observational study. Based on blood androgen concentration, patients with PCOS were divided into HAPCOS (*n* = 69) or non-HAPCOS (*n* = 180) groups.

**Results:**

Although within normal ranges, patients with HAPCOS had significantly (*p* < 0.05) higher peripheral and central systolic blood pressure and pulse pressure, C-reactive protein, low-density lipoprotein cholesterol, triglycerides, glucose, and insulin than subjects with non-HAPCOS, and healthy women. They also had lower N-terminal prohormone of B-type natriuretic peptide (NT-proBNP) concentration. In contrast, their body mass index (BMI) was higher of over 4 kg/m^2^ than patients with non-HAPCOS and nearly 6 kg/m^2^ than in healthy participants. Except for BMI, statistical differences in the cardiometabolic profile were of little clinical relevance.

**Conclusions:**

Young normotensive women with HAPCOS have a worse cardiometabolic profile but lower NT-proBNP concentration than patients with non-HAPCOS. Features of this profile in both PCOS groups are within ranges typical for healthy women. Increased BMI is the only clinically relevant feature differentiating hyperandrogenic from non-hyperandrogenic patients with PCOS, and healthy women.

## Introduction

The polycystic ovary syndrome (PCOS) is an endocrinological disease found in 12–21% of reproductive-aged women with clinical symptoms of oligo/amenorrhea, oligo/anovulatory cycles, hirsutism or male pattern balding, infertility, various psychological symptoms such as depression, anxiety, or psychosexual dysfunction [1]. Metabolic disturbances include obesity, dyslipidemia, insulin resistance (IR), and PCOS, often with overweight or obesity, arterial hypertension, endothelial dysfunction, and atherosclerosis [2–5]. Many clinical symptoms overlap between PCOS and metabolic syndrome, with the latter being a risk factor for developing cardiovascular (CV) diseases and complications.

The CV system has been studied in patients with PCOS for many years, and the definition of PCOS has changed several times. Data on the CV function in patients with PCOS are contradictory—from significant to no involvement at all [6–10].

The N-terminal prohormone of brain natriuretic peptide (NT-proBNP) is an inactive form of the B-type natriuretic peptide. It is secreted by cardiomyocytes in response to increased volume and/or pressure overload, producing myocardial wall stretching. For this reason, it has been used in the diagnosis and monitoring the progress of acute and chronic heart failure. NT-proBNP is also increased in patients with hypertension and ischemic heart disease. In general, the measurement of NT-proBNP is considered a biochemical marker of the CV function [11, 12].

Currently, to diagnose PCOS, two of the following abnormalities are required: oligo/anovulation, polycystic ovaries on ultrasound, and clinical and/or biochemical signs of hyperandrogenism [13, 14]. However, some clinical features of PCOS can be found even in women without hyperandrogenism. Therefore, based on the measurement of androgenic profiling, two opposing phenotypes of PCOS are distinguished: non-hyperandrogenic (non-HAPCOS) and hyperandrogenic (HAPCOS) [15].

Although androgen excess may influence the CV function, including arterial blood pressure (BP) and heart rate (HR) [3, 10, 16, 17], data comparing the CV function between patients with non-HAPCOS and HAPCOS are missing or limited. We assumed that normotensive, young women with PCOS, regardless of hyperandrogenism, have normal CV risk measures despite more unfavorable metabolic parameters than healthy women. In addition, we assumed that significant differences exist between patients with PCOS who are or are not hyperandrogenic in the CV, anthropometric, metabolic and hormonal features.

This study aimed to compare different measures of the CV function, anthropometric, metabolic, and hormonal characteristics in normotensive patients with non-HAPCOS and HAPCOS, and eumenorrheic healthy women.

## Materials and methods

### Participants

The study was conducted at the Department of Endocrinology, Metabolism and Internal Diseases of Poznan University of Medical Sciences. This Department is a regional centre for endocrine diseases for a substantial part of Western Poland.

Patients with suspicion or preliminary diagnosis of PCOS were referred to this Department for more detailed evaluation from several outpatient clinics. Final recruitment was made only by the team of endocrinologists and gynaecologists from this Department. Healthy women were recruited from eumenorheic, overall healthy volunteers who responded to a local newspaper call, and Poznan University of Medical Sciences students. More details on the recruitment criteria are given below.

### Patients with PCOS

Two hundred forty-nine normotensive women with PCOS diagnosed with the Rotterdam criteria, according to recent international guidelines [13, 14], and 85 healthy, eumenorrheic women of comparable age and no overt cardiac or renal disease were studied. We included consecutively recruited normotensive (blood pressure <140/90 mmHg according to the European guidelines) [18] patients with PCOS aged 18–40 years with at least two of the following three features:

- 1. oligoovulation/anovulation;
- 2. hyperandrogenism (elevated androgen concentration: total testosterone (TT) > 2.67 nmol/l, and/or free androgen index (FAI): > 5.5) or hyperandrogenisation (acne, hirsutism);
- 3. the presence of at least 20 follicles in each ovary measuring 2–9 mm in diameter, and/or ovarian volume more than 10 cm^3^ at ultrasound [13, 14, 19, 20]. According to recent international guidelines by Teede et al. [14], in cases of the presence of both irregular menstrual cycles and hyperandrogenism, ultrasound was not necessary for diagnosis. Similarly, ultrasound was not used to diagnose patients within 8 years of menarche [14].

The Ferriman–Gallwey scale assessed the hirsutism score with eight points as the cut-off value. Since clinical signs of acne or hirsutism have other causes than hyperandrogenism, and their assessment can be subjective, we have applied only biochemical criteria of hyperandrogenism.

Diabetes, hypertension, severe liver or kidney disease, the use of oral contraceptive or anti-androgen therapy in the last 3 months, and current pregnancy were exclusion criteria for women with PCOS. We separated two groups with (HAPCOS) and without biochemical hyperandrogenism (non-HAPCOS) from all qualified patients with PCOS.

### Healthy women

Women enrolled in the control group (CG) were regularly menstruating, with no history, symptoms, and signs of hyperandrogenism, diabetes, arterial hypertension, liver or kidney disease, not using any hormonal contraception in the last 3 months. At the time of enrollment, all were euthyroid and not pregnant.

### Laboratory assessment

Blood samples were collected from participants after overnight fasting in the follicular phase of spontaneous menstrual cycles. After separation, serum was frozen −80 ^°^C until assayed.

In both PCOS patients and healthy women, we measured:

- the N-terminal prohormone of B-type natriuretic peptide (NT-proBNP), the Anti-Müllerian Hormone (AMH), luteinizing hormone (LH), follicle-stimulating hormone (FSH), total testosterone (TT), globulin binding sex hormones (SHBG), dehydroepiandrosterone sulfate (DHEAS), estradiol (E2), C-reactive protein (CRP) and insulin using Cobas 6000 Analyzer and dedicated electrochemiluminescence sandwich immunoassays or enzyme linked immunosorbent assays (ELISA) kits (Roche Diagnostics GmbH, Germany).
- Fasting glucose concentration (the hexokinase method, Cobas 6000 Analyzer).
- Total cholesterol (TC), triglycerides (TG), and high-density lipoprotein concentration (HDL) (enzymatic colourimetric method, Cobas 6000 Analyzer). The Friedewald formula calculated the low-density lipoprotein cholesterol (LDL) concentration) [21].
- Androstenedione, dihydrotestosterone (DHT) by the proper ELISA kits (DRG Instruments GmbH, Germany.
- 17-OH-progesterone (17-OHP) by the 17α-OH Progesterone ELISA kit (NovaTec Immunodiagnostica GmbH, Germany).
- glucose and insulin in the oral glucose tolerance test (OGTT) with a load of 75 g of glucose.
- Insulin resistance (IR) derived from the homeostatic model assessment (HOMA) equation [22].
-The free androgen index (FAI) was derived from the formula FAI = 100*(total testosterone*/*SHBG) [23].

### Anthropometric measurements

Body weight and height, waist at the umbilicus level, and hip circumferences around the widest portion of the hips were measured in all subjects. Additionally, body mass index (BMI) and the waist-to-hip ratio (WHR) were calculated according to known equations [24, 25].

### Cardiovascular measurements

In addition to the serum concentration of NT-proBNP (which is proportional to cardiac volume or pressure load), we measured HR, peripheral and central BP.

BP was first measured using the brachial Blood Pressure Monitor (Colin BPM 7000, Colin, Japan) on both right and left arm while the participants were seating for 3 min. The higher reading was taken into further analysis. Next, a piezoelectric tonometer (Colin BPM 7000, Colin, Japan) was placed over their right radial artery for the non-invasive acquisition of the radial (peripheral pressures) arterial pressure waveform in a supine position. This signal was sent to the SphygmoCor Mx device (AtCor Medical, Australia) for the reconstruction of (validated transfer function) of the central pressure waveform typical for the ascending aorta [26–29]. After averaging by the Sphygmocor software, we measured for the peripheral and central pressure waveforms the diastolic BP, systolic BP and pulse pressure (PP). As mean BP and pulse rate are the same at the radial artery and aorta, both were measured once. All patients were in sinus rhythm during monitoring on their ECG; thus, pulse rate equals HR.

### Sample size estimation

In our experience and based on the FAI < 5.5, there are more women with non-HAPCOS than with HAPCOS with the average proportion 2–3:1. Therefore, we used the 3 to 1 ratio for non-HAPCOS vs HAPCOS patients to calculate the sample size. For individuals with PCOS and FAI < 5.5, this index’s average values are between 1.5 and 2.5, depending on the data set, with a standard deviation of ap. 2. For patients with PCOS and FAI > 5.5, the average value, depending on the patients’ studied group, is higher with a larger standard deviation even as high as 6. From our experience, the difference of FAI means between women with FAI < 5.5 and FAI > may be between 2 and 6. Usually, it is 3.5. To estimate the sample size, we used the comparison of two means with the additional assumptions: type I error: 0.01 and type II error: 0.10. The estimated minimum sample size was 146 women with FAI < 5.5 and 49 with FAI > 5.5 (in a total of 195 patients with PCOS). As this study was designed as a prospective one in consecutive patients with PCOS, some of whom might be excluded due to other comorbid conditions, e.g., hypertension or diabetes or use of oral contraceptives, we decided to increase the total number of enrolled patients with PCOS to 270. We enrolled healthy women in a number comparable to the size of HAPCOS group.

### Statistical analysis

Continuous data did not have a normal distribution by the Shapiro–Wilk test, so the descriptive statistics are presented as median and 25–75th. The non-parametric unpaired Mann–Whitney test was used to compare continuous data between all studied groups. For the correlation between CV function and sex hormones, we used the non-parametric Spearman correlation with the presentation of the rho coefficient and *p* values. All tests were two-sided, and only *p* values < 0.05 were considered statistically significant. Statistical analysis was made using MedCalc Statistical Software version 19.0.7 (MedCalc Software bvba, Ostend, Belgium; https://www.medcalc.org; 2019).

## Results

Of all studied patients with PCOS, hyperandrogenism’s biochemical criteria were fulfilled in 69 women (HAPCOS) but not in 180 (non-HAPCOS). The control group consisted of 85 healthy women. Summary statistics for continuous data are shown in Table 1 and for the qualitative data in Table 2.

**Table 1:** Clinical characteristics of all healthy women in the control group (CG), and patients with polycystic ovary syndrome (PCOS) without hyperandrogenism (non-HAPCOS) and with hyperandrogenism (HAPCOS)

Parameter	CG	Non-HAPCOS patients	HAPCOS patients	*P* value for		
	*n* = 85	*n* = 180	*n* = 69	CG vs Non-HAPCOS	CG vs HAPCOS	Non-HAPCOS vs HAPCOS
Age (years)	25.95 (23.09–32.26)	24.86 (22.05–27.84)	23.14 (21.42–27.97)	*0.007*	*<0.001*	*0.20*
Body weight (kg)	60.00 (55.00–67.00)	64.00 (58.00–75.00)	75.00 (61.75–87.25)	*0.013*	*<0.001*	*<0.001*
Height (cm)	167.00 (164.00–170.50)	167.00 (163.00–171.00)	166.00 (162.00–170.25)	*0.687*	*0.314*	*0.44*
BMI (kg/m^2^)	21.46 (19.54–23.67)	22.95 (20.49–26.07)	27.23 (22.28–31.51)	*0.008*	*<0.001*	*<0.001*
WHR	0.85 (0.81–0.89)	0.87 (0.82–0.93)	0.90 (0.85–0.96)	*0.015*	*<0.001*	*0.008*
Creatinine (mg/dl)	0.74 (0.68–0.79)	0.72 (0.65–0.79)	0.76 (0.69–0.82)	*0.265*	*0.573*	*0.101*
CRP (mg/l)	2.40 (0.43–4.18)	0.60 (0.30–1.30)	1.25 (0.45–3.05)	*0.052*	*0.460*	*<0.001*
TC (mg/dl)	168.00 (152.00–183.50)	175.50 (157.50–192.00)	179.00 (157.25–194.75)	*0.056*	*0.057*	*0.686*
HDL (mg/dl)	68.00 (56.00–79.75)	66.00 (57.50–78.00)	59.50 (50.00–67.50)	*0.872*	*0.003*	*<0.001*
LDL (mg/dl)	84.60(73.50–95.08)	89.90 (72.45–108.73)	94.10 (80.65–113.10)	*0.069*	*0.003*	*0.182*
TG (mg/dl)	78.00 (63.00–98.50)	65.00 (50.25–93.50)	84.00 (68.00–119.50)	*0.003*	*0.086*	*<0.001*
Glucose 0 min (mg/dl)	86.00 (83.00–92.00)	86.00 (82.00–90.00)	89.00 (85.00–94.00)	*0.783*	*0.015*	*0.003*
Glucose 120 min (mg/dl)	87.50 (77.00–102.00)	92.00 (82.00–107.00)	104.00 (88.25–116.75)	*0.167*	*<0.001*	*0.002*
Insulin 0 min (uU/ml)	10.00 (7.25–13.00)	8.00 (6.00–12.00)	13.00 (8.75–18.00)	*0.009*	*0.002*	*<0.001*
Insulin 120 min (uU/ml)	31.00 (24.75–47.25)	37.00 (26.00–55.00)	54.00 (34.25–85.25)	*0.324*	*<0.001*	*<0.001*
HOMA-IR	2.05 (1.55–2.78)	1.72 (1.24–2.62)	2.91 (1.87–4.41)	*0.032*	*<0.001*	*<0.001*
TSH (uU/ml)	2.14 (1.65–3.20)	2.03 (1.35–2.88)	2.16 (1.56–2.66)	*0.085*	*0.345*	*0.723*
FSH (mIU/ml)	5.20 (3.20–6.98)	5.85 (4.70–6.90)	5.80 (4.85–6.70)	*0.048*	*0.096*	*0.777*
LH (mIU/ml)	6.60 (3.70–10.70)	8.10 (5.30–12.50)	12.50 (7.25–17.05)	*0.043*	*<0.001*	*<0.001*
LH/FSH	1.32 (0.86–2.32)	1.46 (0.99–2.20)	2.14 (1.41–3.43)	*0.247*	*<0.001*	*<0.001*
17-OHP (ng/ml)	2.05 (1.72–2.85)	2.41 (1.80–2.96)	3.00 (2.40–3.77)	*0.144*	*<0.001*	*<0.001*
AMH (pmol/l)	23.50 (15.00–32.00)	49.00 (38.00–73.53)	62.00 (41.34–95.00)	*<0.001*	*<0.001*	*0.044*
A (ng/ml)	2.52 (1.77–3.15)	3.74 (2.73–4.81)	5.15 (4.08–6.72)	*<0.001*	*<0.001*	*<0.001*
DHEAS (ug/dl)	303.00 (217.75–355.50.)	275.00 (213.00–343.25)	407.00 (313,50–484.50)	*0.509*	*0.074*	*<0.001*
DHT (pg/ml)	292.00 (234.50–354.25)	340.00 (272.00–416.00)	479.50 (398.00–672.00)	*0.103*	*<0.001*	*<0.001*
FAI	1.45 (0.89–2.33)	2.47 (1.69–3.43)	6.86 (5.30–9.97)	*<0.001*	*<0.001*	*<0.001*
SHBG (nmol/l)	71.90 (49.90–120.00)	62.40 (45.90–80.90)	30.60 (22.98–54.50)	*0.207*	*<0.001*	*<0.001*
TT (ng/ml)	0.46 (0.35–0.58)	0.43 (0.35–0.55)	0.78 (0.57–0.89)	*0.371*	*<0.001*	*<0.001*
E2 (pg/ml)	65.50 (34.00–135.00)	40.00 (25.00–67.00)	45.00 (31.75–66.25)	*0.084*	*0.185*	*0.134*
NT-proBNP (pg/ml)	41.58 (29.45–67.74)	39.77 (25.34–63.25)	34.57 (20.00–46.72)	*0.564*	*0.01*	*0.021*
HR (beats/minute)	74.09 (66.81–80.59)	72.43 (66.06–79.10)	73.15 (67.65–84.42)	*0.841*	*0.564*	*0.359*
pSBP (mmHg)	106.97 (100.24–112.51)	105.51 (100.54–111.30)	108.53 (101.63–118.19)	*0.660*	*0.111*	*0.019*
pDBP (mmHg)	63.36 (59.87–68.46)	64.185 (60.70–69.60)	65.33 (60.81–71.45)	*0.666*	*0.296*	*0.286*
pPP (mmHg)	42.43 (37.25–46.25)	41.495 (37.57–45.20)	43.58 (39.79–48.21)	*0.514*	*0.076*	*0.002*
cSBP (mmHg)	93.89 (87.03–97.28)	90.95 (85.85–97.20)	94.87 (88.62–101.39)	*0.443*	*0.246*	*0.026*
cDBP (mmHg)	64.5 (60.98–69.69)	65.135 (61.80–70.30)	66.26 (61.77–73.20)	*0.750*	*0.307*	*0.264*
cPP (mmHg)	26.00 (23.92–29.33)	25.75 (22.67–28.00)	27.3 (25.10–30.37)	*0.165*	*0.153*	*<0.001*
MBP (mmHg)	78.87 (73.36–84.51)	77.16 (73.65–83.71)	80.05 (74.35–86.45)	*0.828*	*0.318*	*0.122*

The results are presented as median and 25–75^th^ percentiles

Laboratory values are expressed using conventional units of measure, with relevant Système International (SI) conversion factors expressed below: creatinine: to get from mg/dL to SI (mmol/L) multiply by 0.0884; CRP: to get from 1 mg/L to SI (nmol/L) multiply by 9.5238 nmol/L; TC: to get from mg/dl to SI (mmol/L) multiply by 0.02586; TG: to get from mg/dL to SI (mmol/L) multiply by 0.01129; glucose: to get from mg/dL to SI (mmol/L) - multiply by 0.0555; insulin to get from uIU/mL to SI (pmol/l) multiply by 6.9444; 17-OHP: to get from ng/ml to SI (nmol/L) multiply by 3.0261; A: to get from ng/mL to SI (nmol/L) multiply by 3.4916 nmol/L; DHEAS: to convert from ug/dL to SI (umol/L) multiply by 0.0271 umol/L; TT: to get from ng/mL to SI (nmol/L) multiply by 3.4672 nmol/L; NT-proBNP: to convert from pg/mL to SI (pmol/L) multiply by 0.1182 pmol/L

*A* androstenedione, *AMH* Anti-Müllerian Hormone, *cDBP* central diastolic blood pressure, *CG* control group, *cPP* central pulse pressure, *CRP* C-reactive protein, *cSBP* central systolic blood pressure, *DHEAS* dehydroepiandrosterone sulfate, *DHT* dihydrotestosterone, *E2* estradiol, *FAI* free androgen index, *FSH* follicle-stimulating hormone, *HDL* high-density lipoprotein concentration, *HOMA* homeostatic model assessment insulin resistance index, *HR* resting heart rate, *LDL* low-density lipoprotein cholesterol, *LH* luteinizing hormone, *MBP* mean blood pressure, *NT-proBNP* N-terminal pro-B-type natriuretic peptide, *pDBP* peripheral diastolic blood pressure, *pPP* peripheral pulse pressure, *pSBP* peripheral systolic blood pressure, *SHBG* globulin binding sex hormones, *TC* total cholesterol, *TG* triglycerides,*TSH* thyroid-stimulating hormone, *TT* total testosterone, *17-OHP* 17-OH-progesterone

**Table 2:** Clinical characteristics of studied patients with PCOS

Criterion	All PCOS *n* = 249	Non-HAPCOS *n* = 180	HAPCOS *n* = 69	non-HAPCOS vs HAPCOS *p* value
Acne	137 (55.2%)	102 (56.7%)	35 (50.7%)	*0.395*
Hirsutism	144 (57.8%)	100 (55.6%)	44 (63.8%)	*0.242*
Polycystic ovaries^a^	185 (74.3%)	128 (71.1%)	57 (82.6%)	*0.064*
BMI ≥ 30 kg/m^2^	47 (18.9%)	24 (13.3%)	23 (48.9%)	*<0.0001*

^a^Volume and the morphology of each ovary, setting the threshold at 10 cm^3^ for increased ovarian volume and 20 for the *2–9* *mm* follicles

### Comparison of patients with PCOS vs healthy women

Patients with PCOS were statistically significantly younger than women from the control group. However, the age difference of 3 years is negligible from a clinical and physiological point of view. Compared with healthy women, patients with PCOS had higher weight, BMI, and WHR, the concentrations of 17-OHP, AMH, androstenedione, TT, and FAI values. The women with non-HAPCOS had lower concentrations of TG, fasting insulin, TSH, and HOMA-IR values than healthy subjects. The concentrations of TT, SHBG, DHEAS, 17-OHP and DHT were comparable in patients with non-HAPCOS vs healthy controls. The patients with HAPCOS had significantly higher concentrations of LDL, DHEAS and DHT, glucose, and insulin before and 120 min after OGTT, and values of HOMA-IR and LH/FSH than healthy women. However, concentrations of SHBG, HDL, and NT-proBNP were lower in individuals with HAPCOS. No differences in HR, mean BP, peripheral and central diastolic BP were found between healthy women and patients with PCOS.

### Comparison of patients with non-HAPCOS and HAPCOS

The patients with HAPCOS had higher weight, BMI, WHR, concentrations of CRP, TG, glucose, and insulin before and 120 min after glucose consumption, LH, 17-OHP, AMH, androgens, values of HOMA-IR, LH/FSH, peripheral and central systolic BP, and PP but lower NT-proBNP and SHBG concentrations. Obesity was fourfold higher in patients with HAPCOS. The values of remaining parameters, including HR, peripheral and central diastolic BP were comparable between women with non-HAPCOS and HAPCOS.

### Association between sex hormones the cardiovascular function

Table 3 shows the results of the non-parametric Spearman correlation between sex hormones or their derivative parameters and the CV function descriptors for pooled data from both the patients with PCOS and control women.

**Table 3:** Spearman correlation between sex hormones or their derivative parameters and descriptors of the CV function in pooled data of patients with PCOS and healthy women

	NT-proBNP		HR		pSBP		pDBP		pPP		cSBP		cDBP		cPP		MBP	
	rho	P value	Rho	P value	rho	P value	rho	P value	rho	P value	rho	P value	rho	P value	rho	P value	rho	P value
FSH	−0.19	<0.001	0.04	0.480	−0.08	0.231	−0.10	0.121	0.002	0.979	−0.09	0.147	−0.10	0.131	−0.004	0.954	−0.103	0.101
LH	−0.19	<0.001	0.14	0.029	−0.04	0.499	−0.09	0.158	0.01	0.782	−0.08	0.221	−0.09	0.179	−0.03	0.607	−0.08	0.221
LH/FSH	−0.08	0.144	0.11	0.077	0.01	0.926	−0.04	0.540	0.03	0.676	−0.03	0.662	−0.04	0.557	−0.02	0.711	−0.03	0.687
17-OHP	−0.11	0.046	0.11	0.089	0.19	0.002	0.16	0.012	0.11	0.070	0.14	0.026	0.16	0.013	0.03	0.588	0.15	0.016
AMH	−0.06	0.288	−0.08	0.227	0.003	0.959	0.01	0.842	−0.06	0.360	−0.01	0.879	0.002	0.980	−0.06	0.332	−0.01	0.866
A	−0.21	<0.001	0.06	0.366	0.08	0.189	0.07	0.246	0.05	0.443	0.05	0.451	0.08	0.227	0.007	0.913	0.06	0.342
DHEAS	−0.18	0.001	0.003	0.956	0.005	0.937	−0.03	0.683	0.05	0.438	−0.01	0.875	−0.02	0.694	0.05	0.409	−0.02	0.762
DHT	−0.22	<0.001	−0.01	0.921	0.10	0.133	0.005	0.941	0.18	0.007	0.07	0.301	0.01	0.908	0.18	0.010	0.04	0.613
FAI	−0.25	<0.001	0.13	0.049	0.18	0.005	0.13	0.058	0.18	0.005	0.17	0.011	0.13	0.043	0.15	0.020	0.15	0.024
SHBG	0.16	0.005	−0.15	0.017	−0.18	0.006	−0.15	0.023	−0.14	0.033	−0.18	0.006	−0.16	0.015	−0.12	0.069	−0.17	0.010
TT	−0.10	0.058	0.05	0.448	0.06	0.375	0.03	0.602	0.09	0.156	0.05	0.431	0.04	0.542	0.07	0.285	0.05	0.444
E2	−0.004	0.944	0.01	0.914	−0.16	0.015	−0.14	0.034	−0.05	0.473	−0.14	0.038	−0.14	0.035	−0.02	0.827	−0.15	0.027

*A* androstenedione, *AMH* Anti-Müllerian Hormone, *cDBP* central diastolic blood pressure, *cPP* central pulse pressure, *cSBP* central systolic blood pressure, *DHEAS* dehydroepiandrosterone sulfate, *DHT* dihydrotestosterone, *E2* estradiol, *FAI* free androgen index, *FSH* follicle-stimulating hormone, *HR* resting heart rate, *LH* luteinising hormone, *MBP* mean blood pressure, *NT-proBNP* N-terminal pro-B-type natriuretic peptide, *pDBP* peripheral diastolic blood pressure, *pPP* peripheral pulse pressure, *pSBP* peripheral systolic blood pressure, *SHBG* globulin binding sex hormones, *TT* total testosterone, *17-OHP* 17-OH-progesterone

The observed correlations were either non-significant or weak, with rho values between 0.14 and 0.25. The concentration of NT-proBNP was negatively correlated with the concentrations of LH, androstenedione, DHEAS, DHT, TT, and FAI values but positively with SHBG. HR was positively correlated with the concentration of LH, the ratio of LH/FSH, FAI, and negatively with the SHBG. Most of the peripheral and central blood pressure descriptors were positively correlated with 17-OH-progesterone, FAI, and negatively with E2 and SHBG. The values of peripheral and central PP were associated with DHT concentration.

## Discussion

The CV system function and risk factors have been extensively studied in PCOS for many years. It is believed that patients with PCOS are at an increased risk for the development of CV diseases. However, the clinical evidence from the long-term follow-up on women’s major CV events with PCOS is either missing or insufficient [3, 7, 30].

This study reports several CV descriptors that describe CV function or are considered predictors of major clinical outcomes in the general population and cardiac patients [31].

We have found no differences in HR and BP between healthy women and normotensive patients from non-hyperandrogenic and androgenic PCOS phenotypes. The concentration of NT-proBNP was comparable between healthy women and subjects with non-HAPOCS. However, patients with HAPCOS had a lower NT-pro-BNP concentration than non-HAPCOS and healthy women. In addition, the women with HAPCOS had higher central and peripheral systolic BP and PP than subjects with non-HAPCOS. Although these differences are statistically significant, their ranges appear to be negligible from the clinical point of view: <5 pg/ml for NT-proBNP, <4 mmHg for central systolic BP, <3 mmHg for peripheral systolic BP, and 2 mmHg for the central and peripheral PP. Besides, values of all measured CV parameters in women with non-HAPCOS and HAPCOS were within normal ranges. In addition, values of NT-proBNP, HR or BP were weakly correlated with sex hormones or their derivative parameters.

Based on the BP profile analysis, the NT-proBNP concentration, an index of increased cardiac load, should be higher in patients with PCOS, but it is not. There is no simple explanation for this finding; data on the NT-proBNP in women with PCOS are limited. Gateva et al. and Deever et al. reported that NT-proBNP concentration was similar in patients with PCOS and healthy women [32, 33]. In both studies, there was no subdivision of PCOS according to the presence of hyperandrogenism. Gateva et al. studied 70 premenopausal women with PCOS and/or obesity and found no differences in NT-proBNP concentrations between obese controls and lean or obese patients with PCOS [32]. Deveer et al. found no difference in NT-proBNP concentration between 25 teenagers with PCOS and 25 regularly ovulating controls [33]. However, Celik et al. found higher NT-proBNP concentrations in 30 patients with PCOS than in 30 age- and BMI-matched controls [34].

We enrolled more patients with PCOS (all of them normotensive) than previous studies and subdivided them into non-hyperandrogenic and hyperandrogenic phenotypes based on the amount of total testosterone and FAI [19, 20]. Such subdivision resulted in a different hormonal and clinical profile of women with non-HAPCOS and HAPCOS going beyond their androgenic hormones.

The patients with HAPCOS, compared to non-HAPCOS individuals, have many features directing them toward the metabolic syndrome and a worse cardiometabolic profile [4]. They have higher concentrations of CRP, TG, fasting and post-OGTT glucose and insulin, HOMA-IR, LDL and lower HDL. They also present with higher resting peripheral and central systolic BP, PP, BMI and WHR, and lower NT-proBNP. The obesity is commoner among the patients with HAPCOS who, on average, have higher WHR of 0.03, body weight of 11 kg and BMI of over 4 kg/m^2^ women with non-HAPCOS.

The increased adipose tissue in patients with HAPCOS seems to be the most plausible explanation for a higher BP co-existence with a lower NT-proBNP concentration. People with excess body weight usually have a faster HR, increased BP, and more often display hypertension [35]. Sodium and water retention, sympathetic and renin-angiotensin-systems activation are increased in obesity and may lead to hypertension development. Natriuretic peptides regulate sodium and water balance and directly interact with the autonomic nervous system and the renin-angiotensin system [36]. Nevertheless, it has been reported that obese people have a reduced concentration of natriuretic peptides compared with leaner individuals [36–40].

Wang et al. studied over 3300 participants from the general population without heart failure and found that both obese men and women had a lower concentration of natriuretic peptides [38]. In another group of 2700 people from the Dallas Heart Study, an observation was made that concentrations of BNP and NT-proBNP decreased with higher BMI, even after adjusting for age, gender, ethnicity, hypertension, left ventricular mass and end-diastolic volume [40]. Similar negative relationships between BMI and natriuretic peptides were confirmed in patients with acute and chronic heart failure [39, 41, 42].

Mechanisms explaining the inverse relationship between the concentration of natriuretic peptides and obesity are uncertain. One of the possibilities is the assumption that obesity is accompanied by a higher glomerular filtration rate and more effective clearing of natriuretic peptides [36]. Another potential explanation comes from observations that adipocytes express natriuretic peptide clearance receptors-C and actively remove BNP from the circulation. Further, hyperinsulinemia attenuates the secretion of natriuretic peptides [43]. Khan et al. studied 3833 individuals from the Framingham Heart Study and 3918 participants from the Malmo Diet and Cancer study. They found a lower NT-proBNP concentration accompanied IR, both in obese and non-obese individuals [43]. The body weight and BMI are higher in our patients with HAPCOS who present hyperinsulinemia and IR.

Chang et al. proposed another explanation. They examined a subgroup of women between 35 and 49 years old from the Dallas Heart Study and reported that free testosterone was negatively correlated with the concentration of BNP and NT-proBNP [44]. They also suggested that since healthy men usually have a lower concentration of natriuretic peptides than women, testosterone might present some inhibitory effect on natriuretic peptides as in our patients with HAPCOS. In women with hypopituitarism and hypoandrogenemia, the prescribed transdermal testosterone diminished the NT-proBNP concentration [45]. Of note, we have observed negative correlations between NT-proBNP and testosterone, A, DHEAS, DHT and the value of FAI, and a positive correlation with SHBG concentration. Our observations are consistent with results from the Framingham Heart Study Third-Generation cohort. In 4056 men and women, NT-proBNP concentration was negatively correlated with free testosterone and positively with SHBG concentrations in both genders [46]. Similarly, Glisic et al. described the presence of a significant link between concentrations of NT-proBNP, T, and DHEAS in postmenopausal women [47].

Finally, natriuretic peptides are also involved in the metabolic processes of lipolysis and energy production [48, 49]. These peptides participate in fat mobilization from white adipose tissue, fat utilization by brown adipose tissue (producing heat), skeletal muscles (fat oxidation), and transforming white to brown adipose tissue. If this is the case, then the low concentration of natriuretic peptides may lead to energy and fat accumulation and, consequently, obesity development as in our subjects with HAPCOS.

So far, links between obesity and the lower concentration of natriuretic peptides have been reported in the general population or cardiac patients [36–40]. Here, we also show the existence of a similar association in patients with PCOS. Probably, a set of abnormalities found in individuals with HAPCOS like the excess of fat tissue, the presence of hyperandrogenism, hyperinsulinemia, and IR, are together responsible for lower NT-proBNP and higher systolic BP and PP in these patients [40, 44, 50–53].

In our study, E2 does not correlate with the NT-proBNP concentration. Nonetheless, data on the relation of estrogens with the natriuretic peptides are conflicting. In some studies, the estrogens administration in premenopausal and postmenopausal women increased the NT-proBNP concentration [54–56], in other investigations, a very weak or no such association was found [44, 47, 57]. Chang et al. reported no differences in BNP and NT-proBNP concentrations between women using or not using oral estrogen in the Dallas Heart Study [44]. Regardless of the lack of correlation of E2 with natriuretic peptides, is inversely correlated with mean BP, and peripheral and central systolic and diastolic BP in our subjects. Estrogens are known for their reducing effects on BP [58–61].

Our study has some limitations. First, we focused only on normotensive (according to European guidelines), women with PCOS [18]. We intended to focus on women who were free of any medications that might influence CV, metabolic and hormonal evaluation. Most of the pharmacological agents used for hypertension treatment show such effects. Further, we based the diagnosis of hyperandrogenism only on biochemical criteria. We resigned from using potential clinical signs such as hirsutism and acne—both hirsutism and acne may have causes other than biochemical hyperandrogenism. Acne can be found quite often and hirsutism occasionally in healthy women [62]. We believe that using strict biochemical criteria of hyperandrogenism is more objective. We used HR, extended peripheral and central blood pressure profile, and the NT-proBNP concentration to evaluate the CV system function. In addition to these CV parameters, some other related to metabolism like the concentrations of fasting or post-OGTT glucose and insulin, CRP, total, LDL and HDL cholesterol or indices such as HOMA-IR, WHR or BMI are known CV risk factors. The findings on NT-proBNP have surprised us. Based on the available literature and several factors potentially reducing the concentrations of natriuretic peptides in people with obesity, we think that NT-proBNP is not a good indicator of the CV function in such individuals, at least when it is in lower concentrations [36–40]. Our study was observational, with measures made only once in each participant and no follow-up data. Therefore, making long-term clinical conclusions based on our results would be speculation only. The final limitation might be a potential bias related to the proportion of selection of patients with PCOS. Our study was performed in a tertiary referral centre for endocrine disease. Whereas this might influence the ratio between the women with non-HAPCOS and HAPCOS, we are convinced it had no effect of other results.

In summary, statistically significant differences between normotensive patients with non-HAPCOS and HAPCOS in blood pressure values NT-proBNP concentration have little clinical meaning in young women with PCOS. Androgens or their derivative parameters correlate with the NT-proBNP concentration but neither with HR nor BP. In contrast, E2 is not associated with NT-proBNP, but it correlates with blood pressure.

With our study, we cannot adjudicate whether the observed differences in CV function and cardiometabolic parameters between patients with non-HAPCOS and HAPCOS are solely caused by hyperandrogenism or overweight/obesity, or both play in a concert. As higher WHR, body weight and BMI are more common in women with HAPCOS than non-HAPCOS, it seems reasonable to assume that potential consequences of obesity such as higher concentrations of glucose, insulin, total and LDL cholesterol and lower HDL cholesterol might be at least initiated and/or amplified by hyperandrogenism. Using everyday speech, we might paraphrase that hyperandrogenism and obesity in patients with PCOS are bad companions.

## Conclusions

In general, normotensive and young patients with PCOS, regardless of their androgenic status, appear to have a normal CV system and metabolic profile function. If hypertension or other abnormalities related to the CV risk and morbidity are found in young women with PCOS, the presence of a clinical problem unrelated to PCOS should be considered.

It should also be kept in mind that the negligible clinical differences between women with non-HAPCOS and HAPCOS were found in very young patients. These differences are present or may be even increase with each day, month and year. If not stopped or reversed, slightly worse CV function and cardiometabolic risk profile might solidify into more considerable clinical differences in future. However, if the small statistically significant differences in the parameters describing the CV function and risk between the non-hyperandrogenic and hyperandrogenic patients with PCOS might translate into future clinical outcomes requires long-term follow-up studies.

## Data Availability

The data that support the findings of this study are available from the corresponding author, P.G., upon reasonable request.

## Code availability

The data that support the findings of this study are available from the corresponding author upon reasonable request.
